# Impact of Interactive Web-Based Education With Mobile and Email-Based Support of General Practitioners on Treatment and Referral Patterns of Patients with Atopic Dermatitis: Randomized Controlled Trial

**DOI:** 10.2196/jmir.2359

**Published:** 2012-12-05

**Authors:** Thomas Schopf, Vibeke Flytkjær

**Affiliations:** ^1^Norwegian Centre for Integrated Care and TelemedicineUniversity Hospital of North-NorwayTromsøNorway

**Keywords:** Atopic dermatitis, Internet, continuing education

## Abstract

**Background:**

The effects of various educational strategies have been examined in continuing medical education. Web-based learning has emerged as an alternative to ordinary classroom lessons.

**Objective:**

To investigate whether an interactive Web-based course including personal guidance via email or cellular phone texting may be used to improve practice behavior of general practitioners in the management of atopic dermatitis.

**Methods:**

General practitioners from all over Norway were eligible for this randomized controlled educational trial. During a period of 6 months, doctors in the intervention group were offered the opportunity to participate in a Web-based course on the management of atopic dermatitis. This was combined with guidance via email or multimedia messaging service (MMS) through mobile phones from a dermatologist. In the control group there was no education or guidance. Main outcome measures were the duration of topical steroid treatment prescribed to patients with atopic dermatitis (primary outcome), number of treatment modalities, and number of referred patients.

**Results:**

We enrolled 46 physicians: 24 doctors were allocated to the intervention group and 22 doctors to the control group. They reported a total of 190 patient treatments. There were no statistically significant differences in the duration of topical steroid treatment or number of treatment modalities between the groups. The lack of effect on the primary outcome may be due to attrition as 54% (13/24) of the participants did not complete the course. 42% (10/24) of physicians sent at least one educational request via email or MMS. While 11% (8/73) of treatment reports in the intervention group were referred to a health care specialist (eg, dermatologist or pediatrician), 30% (21/71) of treatment reports in the control group did so. This difference in the number of referrals was significant (*P* = .03).

**Conclusions:**

A Web-based educational intervention aimed at general practitioners combined with personal support can reduce the number of atopic dermatitis patient referrals to specialists.

## Introduction

Atopic dermatitis (AD) is a common chronic inflammatory skin condition that may affect children as well as adults [1]. In Northern and Western Europe, the prevalence of AD in children was estimated to be 15-25% [2,3], whereas approximately 2-5% of adults were affected [4]. The majority of patients with AD suffer from a mild to moderate form of the disease and is most often treated in primary health care [3,5]. However, general practitioners (GPs) may find the management of patients with AD challenging [6], as guidelines commonly present a wide range of therapeutic modalities [1,7]. For instance, doctors are recommended to identify relevant trigger-factors based on a thorough case history before setting up a specific treatment plan [1,7]. Secondary skin infections are common in AD and warrant special attention [1,7]. Compared to other doctors, dermatologists use more complex treatment regimens including the liberal use of topical steroids [8,9]. In contrast, GPs appear to be more conservative in the use of steroids in terms of potency and treatment duration [6].

The aim of continuing medical education (CME) is to maintain and increase professional competence [10]. A variety of educational strategies and their effects on practice behavior have been examined [11,12]. Web-based CME has emerged as an alternative to ordinary classroom lessons [13]. Benefits include easy access from almost any location, no need for travelling, self-directed and self-paced learning [14]. Studies have shown that Web-based education has similar outcomes compared to traditional face-to-face education [15-18]. Despite participants commonly being physically separated in Web-based education, learners may interact with other learners or teachers through discussion forums or via email [13]. Discussion appears to have a significant effect on knowledge and behavioral change [17].

The aim of this study was to assess whether an interactive Web-based educational intervention may be used to improve practice behavior of GPs in the management of AD patients. The primary outcome was the duration of topical steroid treatment prescribed by GPs. Secondary outcomes were the number of treatment modalities prescribed and the number of referrals to a health care specialist.

## Methods

### Study Design

The study was a randomized controlled educational trial with a two group parallel design and, an allocation ratio of 1:1.

### Participants

Between May 2010 and June 2011 we recruited GPs from all over Norway through advertisements in national medical journals and on the website of the Norwegian Medical Association. All physicians currently employed in general practice were eligible for inclusion. Physicians employed as interns or board certified specialists in dermatology or pediatrics and physicians who previously had participated in our Web-based course were excluded. The study period was 6 months.

### Interventions

"Help, it's itchy!" is a Web-based asynchronous CME course on the management of AD in primary health care. A team of medical experts, web developers, and instructional experts planned and produced the course. The course was designed and delivered via the standard learning management system of the Norwegian Centre of Integrated Care and Telemedicine [19]. The target audience for "Help, it's itchy!" are primary care physicians and nurses. The educational boards of family medicine, dermatology, and pediatrics of the Norwegian Medical Association and the Norwegian Nurses Organization approved the course for CME credits. The course has been held regularly every year since it was launched in 2008. Learners were required to register beforehand in order to get access during the 8 week course period.

**Figure 1:**
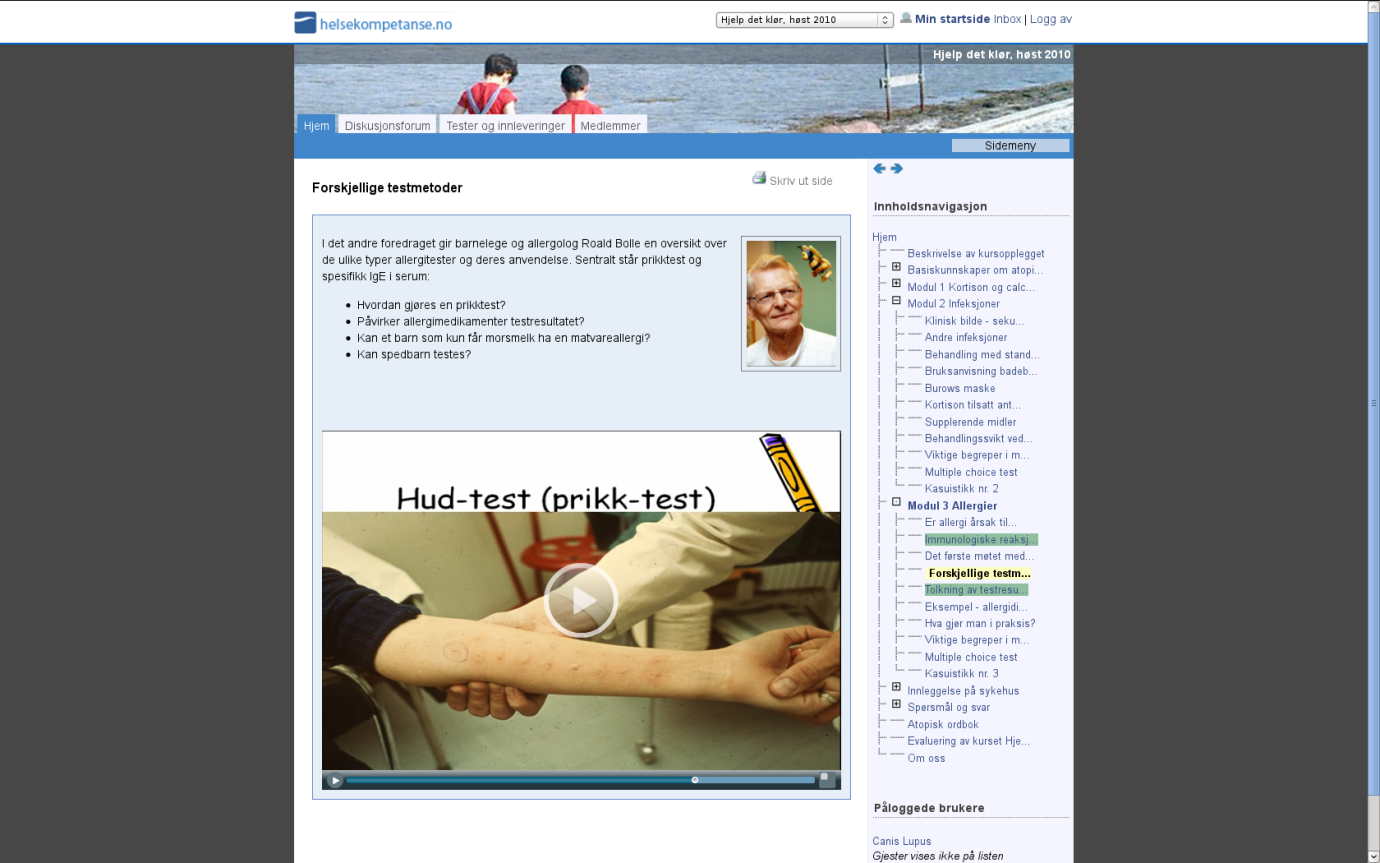
Audiovisual lesson on allergy testing.

**Figure 2:**
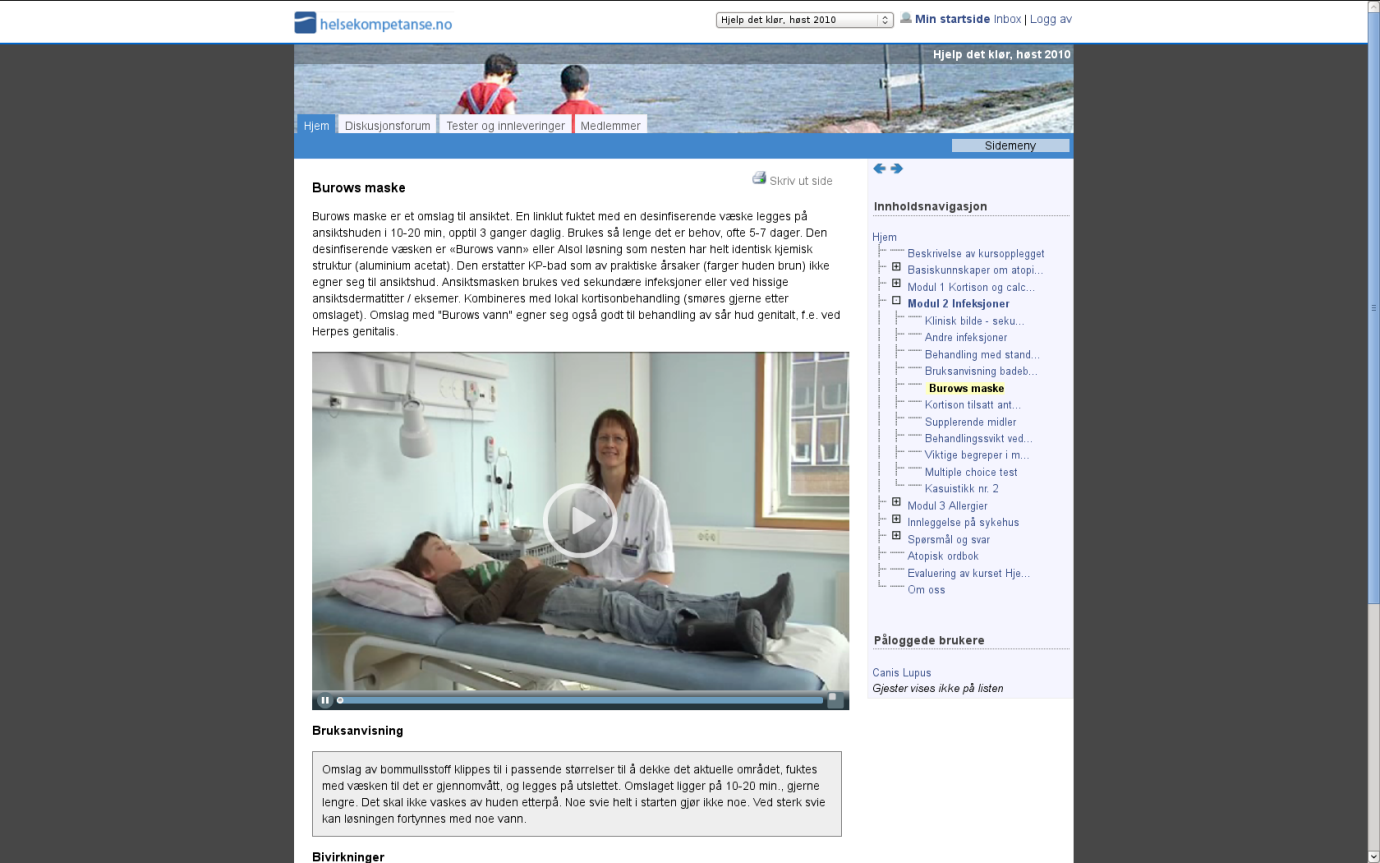
Video showing the use of a facial dressing.

The instructional design of the course was based on the theories of constructivist and experiential learning. The content was presented as narrative text and in audiovisual format (Figure 1). Patient cases were used to explain typical clinical scenarios. Specialist nurses showed how to apply emollients, wet wraps, and facial dressings in 3 instructional videos (Figure 2). Advice on how to deal with cortisone fear was presented in a 7 minute video lecture.

The course was organized into 3 modules (Table 1). Every module contained a set of 8-9 multiple choice questions for self-assessment. Learners received automatic feedback on the screen immediately after completion of the test set. In every module there was also a homework assignment containing a clinical case. Photographs of eczema skin changes were provided in the assignments of module 1 and 2 for better understanding. Learners were asked to present a treatment plan for each case. Physicians who wished to receive CME credits had to submit and pass the homework assignments within the first 6 weeks after initial login. The course instructor (author TS) provided learners with detailed, personalized feedback on the assignments 5-7 days after submission.

Learners were free to discuss with other learners and the course instructor in a forum. In addition, the instructor was accessible via email or multimedia messaging service (MMS).

**Table 1:** Course content.

Section	Topics
Introduction	Etiology; natural history; diagnosis; skin care; management of pruritus.
Module 1 Steroids and calcineurin inhibitors	Use of steroids on various body sites; dosage and tapering-off; side effects; maintaining control; steroid fear; calcineurin inhibitors.
Module 2 Infections	Features of infected eczema; differential diagnosis of infections; procedures for topical treatment; treatment failure.
Module 3 Allergies	Diagnosis of allergies; testing.
Appendix	Specialist treatment; phototherapy.

### The Intervention Group

Physicians allocated to the intervention group were offered to participate in the Web-based course "Help, it's itchy!" including personal guidance via email or MMS on their cellular phone. They were registered for the online course and received information regarding access to the course including a username and password. There was unlimited access to the Web-based curriculum for the entire study period starting 1-3 days after randomization. Physicians in the intervention group were free to send educational requests via email or MMS to the course instructor (author TS) during the entire study period. While the Web-based course was focusing on the treatment of AD, physicians were encouraged to send requests about all topics within the field of AD via email or MMS. They were specifically offered to discuss real cases from their practice and could attach close-up photographs showing the patient's skin lesions provided that the patient had given informed consent. The course instructor responded to requests within 1-2 working days by sending an answer via email or MMS. The requests were for educational purposes only. Physicians were instructed to ensure that no data or images that could possibly lead to the identification of the patient were transferred. They were informed prior to the study that sending requests was not possible for the referral of patients to specialist health care.

### The Control Group

Physicians in the control group neither had access to the Web-based course nor could they send educational requests via MMS or email. After the 6 month trial period ended, we offered all physicians in the control group to continue in a second trial phase. They were offered to participate in a subsequent Web-based course but without the option to send requests via email or MMS. A follow-up questionnaire was sent to these doctors 4 weeks after completing the Web-based course.

### Data Collection

Physicians in both groups were requested to fill in a short online survey (Multimedia Appendix 1 and 2) reporting their treatment prescriptions every time a patient consulted them with AD during the 6 month study period. For the purpose of this study we defined a patient with AD as a person of any age with a clinical diagnosis of AD, or a person probably having AD as judged by the participating physicians. In addition, physicians were asked to fill in online questionnaires about working experience, attitudes and habits regarding the management of AD at start-up and at the end of the study period. In the intervention group there were also questions concerning satisfaction of sending educational requests via email or MMS. Doctors were asked to rate their agreement on 4 statements concerning satisfaction by the use of a Likert-type rating scale containing 5 levels.

The start-up questionnaire and one treatment survey had to be submitted before randomization. Physicians were not required to report treatments immediately after they had seen the patient but were advised to do this at the end of the working day. A reminder message was regularly sent by email every 3 weeks to all participants. The online form used to collect data on the treatments had multiple-choice questions. Physicians were asked to report the number of days they had instructed the patient to use steroid creams or ointments, including tapering. Numerous treatment modalities were listed on the form and doctors had to check off which modalities they had prescribed. Treatment modalities included were emollients, baths, dressings, topical steroids (specifying potency class I-IV: I mild, IV very potent), topical calcineurin inhibitors, wet wrap dressings, oral antihistamines, oral antibiotics, oral steroids and dietary eliminations. Finally there were questions about referral to specialist health care. The physicians were asked to indicate whether they intended to refer the patient and if so, to specify the reasons for referral and to which specialty. Reasons for referral included uncertainty about the diagnosis, flare of the disease, poor response to treatment, need for allergological investigation, and other reasons. We made no attempt to collect data on the severity of AD because we considered it unrealistic to train participants in using a validated scoring algorithm for AD.

After collecting the data according to the study protocol, we also performed a content analysis of the educational requests sent via email or MMS. Common themes were identified and grouped accordingly. The authors TS and VF did the content analysis independently. Disagreement was resolved by consensus.

### Sample Size

The design of the trial was based on a significance level of 5% and a power of 80% against a difference of 3 days (SD=4) in the duration of topical steroid treatment between the groups. This difference appeared meaningful based on our clinical experiences. In calculating the sample size we had to consider the number of treatment reports that each participating physician was going to submit. Assuming an average of 4 measurements per physician, 20 participants would be required in order to show a statistically significant difference in the primary outcome. In the case of only one treatment report per physician, 59 participants would be required. Since the number of measurements per participant was difficult to estimate prior to the trial, we aimed at reaching a sample size of 59 participants. Allowing for a 20% drop out rate, 74 participants had to be enrolled.

### Randomization

Randomization was arranged consecutively from September 2010–June 2011 via the central telephone randomization service at the Clinical Research Department of the University Hospital of North-Norway. We decided on permuted-block randomization to avoid uneven group sizes. As the severity of AD (and consequently the practice behavior of the participants) may be influenced by seasonal climatic variations, bias could be introduced when more treatments were reported in one of the groups during a specific season (eg, winter). Randomization lists were computer generated using block randomization with random block sizes 4, 6, and 8. The investigators were blinded to the block sizes. Participants were informed by email to which group they had been allocated and started in the trial immediately.

### Statistics

Data were analyzed on an intention-to-treat basis. We used a generalized estimating equations model in all outcome analyses to account for random effects introduced by doctors reporting more than one treatment during the study period. An exchangeable covariance structure handled treatment data as within-subject repeated measurements. All data analyses were performed using the IBM SPSS 19 program (IBM, New York, USA).

### Ethical Considerations

The Regional Committee for Medical and Health Research Ethics in Northern Norway (REK-Nord) reviewed the study protocol and concluded that the study did not need approval as this was a non-clinical trial that did not investigate health outcomes. For the same reason, the study was not included in a clinical trials registry (Editorial note: JMIR published this trial despite failure to register, as, according to the International Committee of Medical Journal Editors, registration is not necessary if the purpose of a trial is to examine the effect on health care providers). The protocol (in Norwegian) can be downloaded from the Internet [20]. All physicians gave informed consent before enrolment.

## Results

**Figure 3:**
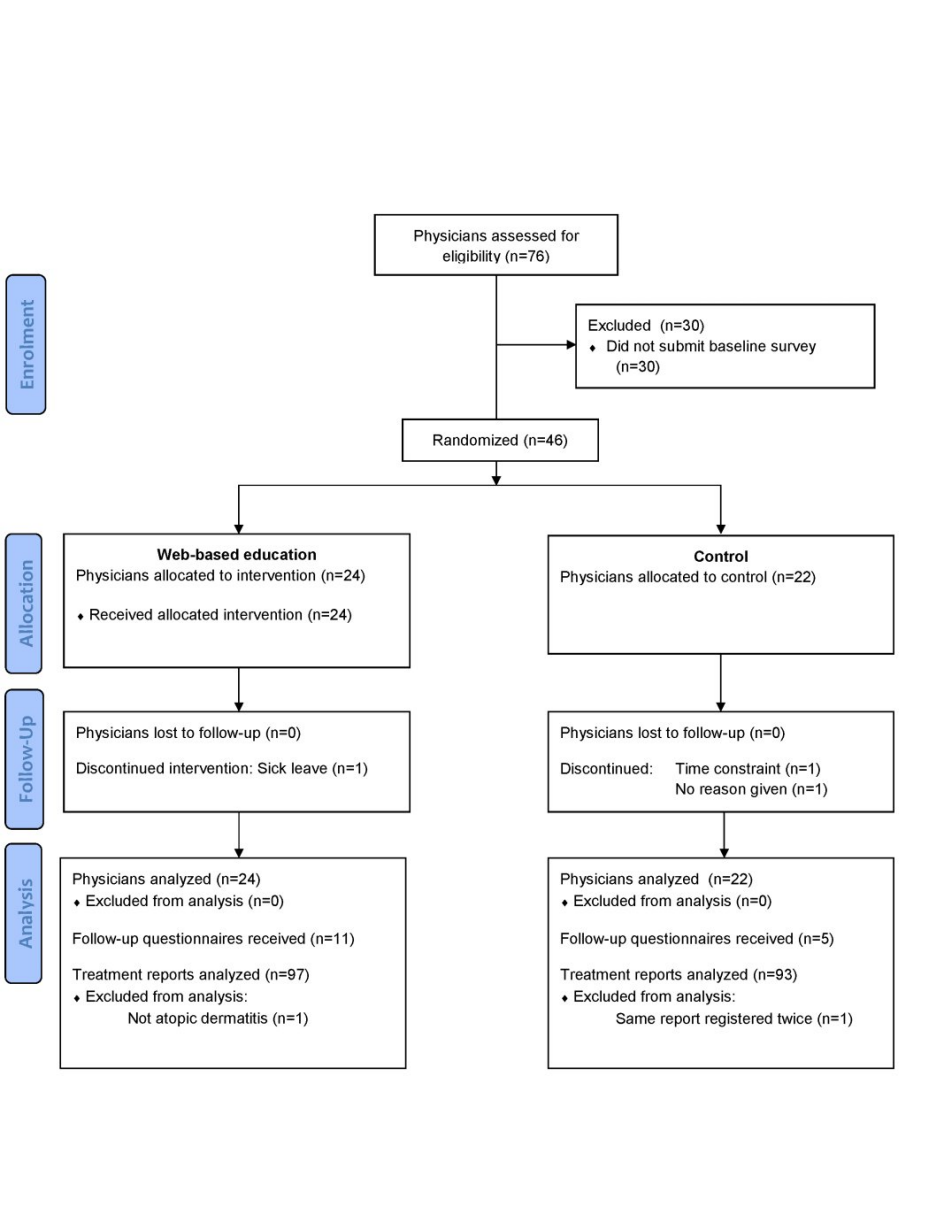
Flow diagram.

Overall, 76 general practitioners were eligible for the study (Figure 3). At the end of the recruitment phase, 46 physicians had submitted a full set of questionnaires. The intervention group consisted of 24 physicians and the control group had 22 physicians. Baseline demographic data for enrolled physicians are shown in Table 2.

The doctors reported a total of 190 patient treatments including baseline data (intervention group: 97 treatments, control group: 93 treatments). Overall 35.4% (67/189) of the treatments were related to adult patients (intervention group: 34/97, 35.1%; control group: 33/92, 35.9%). Treatment reports were submitted on average 10.2 (SD=6.8) weeks after randomization (range 1-26). A summary of reported treatments at baseline is shown in Table 3. The enrolled physicians were representing 43 health centers from all over Norway.

The duration of topical steroid treatment prescribed by the physicians is shown in Table 4. There was no significant difference between the groups (*P*=.82). However, there was a significant increase in the duration of topical steroid treatment compared to baseline for both groups (*P*=.02). The mean number of treatment modalities prescribed at baseline was 2.3 in both the intervention and control group (SD= 1.0 and 0.9 respectively). During the study period physicians in the intervention group prescribed on average 2.3 modalities (SD=1.0) and for physicians in the control group we found 2.0 modalities (SD=0.9). This difference was neither significant between the groups (*P*=.19) nor compared to baseline (*P*=.27). Details of the treatment modalities reported are presented in Table 5.

Overall, 15 doctors (intervention group: 7, control group: 8) reported at least one referral during the study period. 11% (8/73) of treatment reports in the intervention group indicated referral to specialist health care, whereas 30% (21/71) of treatment reports in the control group did so. The difference in the number of referrals was significant (Wald *χ* ^*2*^_*1*_= 4.70, *P* = .03). For details of the referrals see Table 6.

While 63% (15/24) of physicians in the intervention group had logged into the course website at least once, 46% (11/24) of physicians completed the course and received CME credits. A total of 32 educational requests were received via email or MMS. 42% (10/24) of physicians had sent at least one educational request via email or MMS. 29% (7/24) of physicians had neither logged into the course website nor sent any educational requests via email or MMS. Three postings were made in the discussion forum on the course website. Table 7 shows results concerning satisfaction of sending requests via email or MMS. Common themes identified in the educational requests are presented in Table 8.

As only 5 physicians in the control group submitted follow-up questionnaires, the planned comparison of follow-up questionnaires in the two groups was omitted.

**Table 2:** Characteristics of enrolled physicians (N=46).

		Overall	Control	Intervention	Test of significance
**All physicians**		100% (46/46)	48% (22/46)	52% (24/46)	
	Male	43% (20/46)	41%^a^ (9/22)	46%^a^ (11/24)	*X* ^*2*^= 0.002; *P*= .97
	Female	57% (26/46)	59%^a^ (13/22)	54%^a^ (13/24)	
**Working experience**					
	Mean (years)	7.5	6.1	8.8	*F*= 1.97; *P*= .16
	Range	1-27	1-27	1-25	

^a^ Percentage within groups

**Table 3:** Baseline data of reported treatments (N=46).

		Overall	Control^b^	Intervention^b^
**Treatment modalities**				
Topical steroid		96% (44/46)	96% (21/22)	96% (23/24)
Topical steroid class^a^				
	I	34% (15/44)	27% (6/22)	38% (9/24)
	II	34% (15/44)	41% (9/22)	25% (6/24)
	III	30% (13/44)	27% (6/22)	29% (7/24)
	IV	2% (1/44)	0% (0/22)	4% (1/24)
Potassium permanganate bath		4% (2/46)	5% (1/22)	4% (1/24)
Burow's solution wet dressing		0% (0/46)	0% (0/22)	0% (0/24)
Wet wrap dressing		4% (2/46)	0% (0/22)	8% (2/24)
Elimination diet		15% (7/46)	14% (3/22)	17% (4/24)
**Referred**		22% (10/46)	23% (5/22)	21% (5/24)

^a^N=44

^b^Percentage within groups

**Table 4:** Duration of topical steroid treatment (N=150).

	Control^a^	Intervention^a^
Baseline	16.0 (SD=7.1)	15.7 (SD=7.1)
Study period	19.3 (SD=9.9)	20.6 (SD=11.3)

^a^ Mean number of days

**Table 5:** Treatment modalities used (N=144).

	Overall	Control^a^	Intervention^a^
Emollients	79.2% (114/144)	78% (55/71)	81% (59/73)
Topical steroid	83.3% (120/144)	83% (59/71)	84% (61/73)
Potassiumpermanganate bath	9.7% (14/144)	3% (2/71)	16% (12/73)
Burow's solution wet dressing	3.5% (5/144)	1% (1/71)	6% (4/73)
Calcineurin inhibitor	5.6% (8/144)	6% (4/71)	6% (4/73)
Wet wrap dressing	4.9% (7/144)	3% (2/71)	7% (5/73)
Oral antihistamine	13.9% (20/144)	14% (10/71)	14% (10/73)
Oral antibiotic	1.4% (2/144)	0% (0/71)	3% (2/73)
Oral steroid	3.5% (5/144)	3% (2/71)	4% (3/73)
Elimination diet	7.6% (11/144)	6% (4/71)	10% (7/73)

^a^ Percentage by study group

**Table 6:** Referral characteristics (N=29).

		Overall	Control^a^	Intervention^a^
**Referred**		20.1% (29/144)	30% (21/71)	11% (8/73)
	To dermatologist	83% (24/29)	86% (18/21)	75% (6/8)
	To pediatrician	17% (5/29)	14% (3/21)	25% (2/8)
**Reason** ^b^				
	Diagnosis uncertain	35% (10/29)	33% (7/21)	38% (3/8)
	Flare	35% (10/29)	33% (7/21)	38% (3/8)
	Treatment failure	41% (12/29)	38% (8/21)	50% (4/8)
	Investigation of allergies	35% (10/29)	38% (8/21)	25% (2/8)
	Other reasons	10% (3/29)	14% (3/21)	0% (0/8)

^a^Percentage within groups

^b^Several reasons possible

**Table 7:** Satisfaction with sending requests (N=9).

	Mean score^a^	Range
Sending requests was easy	4.5	4-5
The advice given was useful	4.7	4-5
Wish for similar service in other specialties	4.8	4-5

^a^1=strongly disagree; 5=strongly agree

**Table 8:** Common themes in the educational requests^a^ (N=32).

**General questions** (not related to a case)		25% (8/32)
**Discussion of a case**		91% (29/32)
	Diagnosis	19% (6/32)
	Feedback on treatment given	63% (20/32)
	What to do next	50% (16/32)
	Referral	9% (3/32)

^a^Several entries for each request possible

## Discussion

The educational intervention in our trial combined a Web-based course with the possibility to discuss both general issues and concrete cases from the GPs' own practices with a dermatologist. The main findings are that physicians in the intervention group referred fewer patients to secondary health care and that there were no differences between the groups in the duration of topical steroid treatment and the number of treatment modalities prescribed. The reasons for referral appeared to be similar in both groups. Treatment failure and flare were the reported reasons for referral in more than half of the cases.

In Northern and Western Europe, most patients with AD are treated in primary health care [3,5]. This is in line with the intention of policy makers who wish to move chronic care away from hospitals and into the communities [21]. Patients with severe AD, uncertain diagnosis, treatment failure, or complications may require referral to a specialist [7]. However, according to the literature, the majority of referred AD patients had mild to moderate disease [5,9,22]. Because of the high prevalence of AD, even a small reduction in referral rates may have a considerable impact on the workload of dermatologists and pediatricians dealing with AD patients. Reducing referrals may also have economical consequences. In 2010 there were 5406 hospital-based outpatient consultations with children with AD registered in Norway [23]. Every consultation was reimbursed with at least 273 NOK [23]. In contrast, the reimbursement in primary health care for a similar consultation was 136 NOK [24]. Based on a 20% reduction in referrals and a potential saving of 137 NOK per referral [23,24], there would be a national annual saving of 148 124 NOK. The development of Web-based education is costly, but may still be cost-efficient in the long run [15]. Future research on the cost effectiveness of educational interventions should also consider possible changes in referral behavior.

Our data suggest that a Web-based educational intervention aimed at primary care physicians may help to reduce referrals of AD patients. In a review, Akbari and coworkers reported that educational activities led by secondary care providers had a significant effect on referral behavior [25]. In contrast, the passive dissemination of guidelines appeared ineffective [25].

It seems that some topical treatment modalities, for example potassium permanganate baths and Burow's solution dressings, were used more frequently in the intervention group compared to the control group. The use of class I steroids was lower in the intervention group, whereas class III steroids were more frequently used. But there was no significant difference in the secondary endpoint, the mean total number of treatment modalities.

Concerning the duration of topical steroid treatment, the primary outcome of the trial, there was no significant difference between the groups. Regarding sample size, the number of participants appeared sufficient to show a difference in the primary outcome. On average, every doctor in our trial submitted 4.1 (SD=3.5) treatment reports. According to the assumptions we made when sample size was calculated, 20 doctors would be required in the trial.

The lack of effect on the primary outcome may be due to attrition as half of the participants did not complete the course. On the other hand, more than two thirds of the participants used the intervention at least once.

However, we found a significant increase in the duration of topical steroid treatment as compared to baseline for both groups. This might be a Hawthorne effect [26,27]: it seems possible that the awareness of being studied may have influenced the participants' behavior. The doctors in both groups were fully aware of being part of an investigation. They also probably understood that topical steroid therapy was under investigation since several questions in the survey addressed this topic. It is possible that this awareness influenced the behavior-doctors in both groups were keen to follow current treatment guidelines. However, since the exact mechanisms behind Hawthorne effects are unknown, it seems difficult to draw any firm conclusions regarding their influences on the participants [26,27].

Another possible explanation for the increase in steroid treatment duration might be the online form used for the collection of data. On the form, various treatment options were listed. Repeatedly using this form, physicians in both groups may have realized shortcomings in their knowledge of the management of AD. This might have stimulated physicians to reflect and learn which, in turn influenced treatment in both groups.

There are certain limitations in this study. First, it is likely that some GPs did not report all of their treatments during the study period. On the other hand, it seems unlikely that missed patients would have different effects across the two groups.

Second, doctors who enrolled in the trial possibly had a more positive attitude towards Web-based education. Other physicians may still perceive barriers to engage in eLearning and the applicability of our results may therefore be limited. There are currently no other Web-based dermatology courses in Norwegian, but the national CME program contains a variety of online courses in different specialties [28]. Furthermore, online course materials are now being used by many medical schools [29] and we believe that in the future nearly all physicians will become familiar with the use of Web-based educational activities [30].

Finally, our data are based on a 6 month study period. We do not know the effects of our intervention from a long-term perspective. The educational intervention might not affect the total number of referrals in the long run but rather just postpone them. This needs further investigation.

We believe that our findings are applicable to other medical fields within a general practice setting. More than two thirds of the physicians in the intervention group used either the Web-based course or sent educational requests for guidance via email or MMS. The instructional methods used in the course may suit other CME courses in general practice.

In conclusion, as many AD patients who are referred to specialist health care have only mild to moderate disease, there seems to be a potential to reduce unnecessary referrals [5,9,22]. Our study suggests that a Web-based educational intervention aimed at primary care physicians may help reach this goal.

## Multimedia Appendix 1

Online questionnaire used to collect treatment data (in Norwegian).

## Multimedia Appendix 2

Translation of questionnaire.

## Multimedia Appendix 3

CONSORT-EHEALTH Checklist V.1.6.1 [31].

## Abbreviations

AD: atopic dermatitis
CME: continuing medical education
GP: general practitioners
MMS: multimedia messaging service
