# Supplementary material for: Impact of Interactive Web-Based Education With Mobile and Email-Based Support of General Practitioners on Treatment and Referral Patterns of Patients with Atopic Dermatitis: Randomized Controlled Trial
Source: J Med Internet Res. 2012 Dec 5;14(6):e171. doi: 10.2196/jmir.2359 (PMC3849841; doi:10.2196/jmir.2359)
Supplement: Supplementary file 1 [file jmir_v14i6e171_app1.pdf]

Spørsmål 1 /

Er pasienten et barn (dvs under 18 år) eller voksen?

- ✗ ☐ Barn
- ✗ ☑ Voksen

Spørsmål 2 /

Hvilken behandling har du gitt pasienten? (Flere mulig)

- ✓ ☑ Fuktighetskrem
- ✗ ☐ Kalibad
- ✗ ☐ Omslag inkludert Alsol/Burows ansiktsmaske
- ✗ ☐ Kortisonkrem / salve
- ✗ ☐ Protopic / Elidel
- ✗ ☐ Våtbandasje / annen okklusjon
- ✗ ☐ Antihistamin
- ✗ ☐ Systemisk antibiotika
- ✗ ☐ Systemisk kortison
- ✗ ☐ Eliminasjon av matvarer

Spørsmål 3 /

Dersom du krysset av på kortisonkrem / salve i spørsmål 2, hvilken type kortison var det du ga? (Flere mulig)

- ✗ ☐ Gruppe 1
- ✗ ☐ Gruppe 2
- ✗ ☐ Gruppe 3
- ✗ ☐ Gruppe 4
- ✗ ☐ Vet ikke

Spørsmål 4 /

Dersom du krysset av på kortisonkrem / salve i spørsmål 2: Hvor lenge skal pasienten bruke kortisonkrem / salve? Angi antall dager med kortisonbehandling inkludert tiden for evt. nedtrapping.

Spørsmål 5 /

Skal du henvise pasienten til spesialist?

- ✓ ☑ Nei.
- ☐ Ja, til barnelege.
- ☐ Ja, til hudlege.

Spørsmål 6 /

Dersom du skal henvise pasienten til spesialist, hva er årsakene? (Flere mulig)

- ✗ ☐ Usikkerhet omkring diagnosen
- ✗ ☐ Tilstanden har blusset opp
- ✗ ☐ Behandlingen fungerer dårlig
- ✗ ☐ Ønsker allergiutredning / testing
- ✗ ☐ Annet

Spørsmål 7 /

Evt. kommentarer til behandlingen.
