# Supplementary material for: Impact of Interactive Web-Based Education With Mobile and Email-Based Support of General Practitioners on Treatment and Referral Patterns of Patients with Atopic Dermatitis: Randomized Controlled Trial
Source: J Med Internet Res. 2012 Dec 5;14(6):e171. doi: 10.2196/jmir.2359 (PMC3849841; doi:10.2196/jmir.2359)
Supplement: Supplementary file 2 [file jmir_v14i6e171_app2.pdf]

Question 1

Is the patient a child (ie, less than 18 years old) or adult?

_ Child
_ Adult

Question 2

What kind of treatment did you prescribe to the patient? (several options possible)

_ Emollients
_ Potassiumpermanganate bath
_ Dressings (including Burow's facial dressing)
_ Topical steroid cream/ointment
_ Protopic / Elidel (calcineurin inhibitors)
_ Wet-wraps
_ Antihistamine
_ Oral antibiotics
_ Oral steroids
_ Elimination diet

Question 3

In case you ticked “Topical steroid cream / ointment“ in question 2, of which potency was the prescribed topical steroid?

_ Class 1
_ Class 2
_ Class 3
_ Class 4
_ Don't know

Question 4

In case you ticked “Topical steroid cream / ointment“ in question 2, for how long should the patient use topical steroids? Please state the number of days of recommended steroid therapy including tapering-off.

___

Question 5

Are you refering the patient to a specialist?

_ No.
_ Yes, to a pediatrician.
_ Yes, to a dermatologist.

Question 6

In case you are refering the patient to a specialist, what is the reason for referral?

_ Uncertainty about the diagnosis
_ Flare-up
_ Poor treatment effect
_ Allergy investigation needed
_ Other

Question 7

Additional comments on the treatment.

___
